# Teleangiectatic Osteosarcoma Treated by Surgery and Chemotherapy: A Report of 223 Affected Patients From the Cooperative Osteosarcoma Study Group (COSS)

**DOI:** 10.1002/cam4.71211

**Published:** 2025-09-09

**Authors:** Stefan S. Bielack, Vanessa Mettmann, Daniel Baumhoer, Andreas Beilken, Claudia Blattmann, Godehard Friedel, Jendrik Hardes, Wolf Hassenpflug, Leo Kager, Matthias Kevric, Thekla von Kalle, Andreas Kulozik, Markus Metzler, Michaela Nathrath, Claudia Rossig, Benjamin Sorg, Mathias Werner, Stefanie Hecker‐Nolting

**Affiliations:** ^1^ Pädiatrie 5 (Onkologie, Hämatologie, Immunologie), Stuttgart Cancer Center, Zentrum für Kinder‐, Jugend‐ und Frauenmedizin Klinikum Stuttgart – Olgahospital Stuttgart Germany; ^2^ Klinik für Kinder‐ und Jugendmedizin, Pädiatrische Hämatologie und Onkologie Universitätsklinikum Münster Münster Germany; ^3^ Klinik für Kinder‐ und Jugendmedizin – Hämatologie und Onkologie Universitätsmedizin Mannheim Mannheim Germany; ^4^ Knochentumor‐ Referenzzentrum, Institut für Medizinische Genetik und Pathologie Universitätsspital Beider Basel, und Basel Research Centre for Child Health Basel Switzerland; ^5^ Kinder‐ und Jugendklinik, Städtisches Klinikum Braunschweig gGmbH Braunschweig Germany; ^6^ Klinik für Pädiatrische Hämatologie und Onkologie Medizinische Hochschule Hannover Hannover Germany; ^7^ Universitätsklinik für Thorax‐, Herz‐ und Gefäßchirurgie – Sektion Thoraxchirurgie Universitätsklinikum Tübingen Tübingen Germany; ^8^ Klinik für Tumororthopädie und Sarkomchirurgie Universitätsklinikum Essen Essen Germany; ^9^ Klinik und Poliklinik für Pädiatrische Hämatologie und Onkologie, Zentrum für Geburtshilfe, Kinder‐ und Jugendmedizin Universitätsklinikum Hamburg Eppendorf Hamburg Germany; ^10^ St. Anna Kinderspital Universitätsklinik für Kinder‐ und Jugendheilkunde der Medizinischen Universität Wien Vienna Austria; ^11^ St. Anna Children's Cancer Research Institute (CCRI) Vienna Austria; ^12^ Radiologisches Institut (Kinderradiologie), Zentrum für Kinder‐, Jugend‐ und Frauenmedizin, Klinikum Stuttgart – Olgahospital Stuttgart Germany; ^13^ Abteilung für Pädiatrische Onkologie, Hämatologie und Immunologie Universitätsklinikum Heidelberg Heidelberg Germany; ^14^ Kinder‐ und Jugendklinik – Pädiatrische Onkologie und Hämatologie Universitätsklinikum Erlangen Erlangen Germany; ^15^ Klinik für Pädiatrische Hämatologie, Onkologie, Psychosomatik und Systemerkrankungen Klinikum Kassel Kassel Germany; ^16^ Kinderklinik der TU München‐Schwabing Munich Germany; ^17^ Vivantes Netzwerk für Gesundheit GmbH, Fachbereich Pathologie, Osteopathologie – Referenzzentrum Berlin Germany

**Keywords:** chemotherapy, demographics, osteosarcoma, outcomes, surgery, teleangiectatic

## Abstract

**Purpose:**

Teleangiectatic osteosarcoma is a histologic subtype of osteosarcoma that can mimic aneurysmal bone cysts and has so far been incompletely characterized.

**Patients and Methods:**

We used the database of the Cooperative Osteosarcoma Study Group COSS (patient‐registration 1980–2019) to better understand this rare histologic variant.

**Results:**

223 eligible patients were identified, 164 having reference pathology (median age 15.9 (3.7–69.7) years; male 134, female 89; tumor sites limb 208 (201 metaphyses, 66 pathologic fractures), trunk 13, head & neck 2; 26 with primary metastases). Known tumor‐predisposition syndromes were rare. Therapy included surgery in 215, radiotherapy in 10, and chemotherapy in all patients. Tumor response to preoperative treatment was good in 71% of 165 cases with available data. After a median follow‐up of 7.1 (0.2–32.1) years for all patients and 10.5 (0.2–32.1) years for 152 survivors, 5‐/10‐year actuarial event‐free and overall survival expectancies were 61%/57% and 73%/66%, respectively. Five unrelated malignancies occurred during this period. The presence of primary metastases, pathologic fracture, poor response to neoadjuvant chemotherapy, and not obtaining a complete macroscopic remission were associated with inferior outcomes for both event‐free and overall survival (*p* < 0.01).

**Discussion:**

This large analysis proves teleangiectatic osteosarcoma to be a disease predominantly of the metaphyses of the young. While detected only rarely, the true incidence of genetic tumor predispositions would require prospective assessments. Behaving like other osteosarcoma subtypes in many other ways, this variant may show greater chemosensitivity and hence somewhat better outcomes than other subtypes.

## 1 Introduction

Teleangiectatic osteosarcoma represents one of the fully malignant subtypes of conventional osteosarcoma currently recognized by the World Health Organization (WHO) [1]. Histopathologically, it is characterized by cystic spaces which may be empty or blood‐filled. These are lined by highly atypical neoplastic cells often exhibiting atypical mitoses. While rather scarce in some tumors, osteoid formation by the neoplastic cells is a *sine qua non* for making this diagnosis [1, 2].

Like other types of high‐grade osteosarcoma, the teleangiectatic variant is associated with an extremely high risk of systemic spread if left untreated. Metastases typically affect the lungs and, less frequently, distant bones or other sites. Prognosis without chemotherapy is considered just as grave as that of osteosarcoma in general [2].

Teleangiectatic tumors make up few of all osteosarcomas. In the large EURAMOS study of over 2000 osteosarcomas, for instance, their proportion was only 4% [3]. The number of large, dedicated analyses from the era in which chemotherapy became routine is therefore limited. To our knowledge, the largest reports include a series from the Rizzoli Institute [4], an epidemiologic paper utilizing the Surveillance, Epidemiology, and End Results (SEER) dataset [5], and a subgroup of all patients entered into the international EURAMOS study [3]. These include 87, 127, and 95 eligible patients, respectively. Most patients in a fourth relatively large series stem from many decades ago when treatment was purely surgical [2]. Hence, knowledge about this tumor variant when treated under current conditions remains limited.

The Cooperative Osteosarcoma Study Group COSS has been gathering information on osteosarcomas for more than four decades [6]. This allows us to investigate even the infrequent subtypes of this disease in considerable detail. We therefore now report on patient‐ and tumor‐related data associated with teleangiectatic osteosarcoma, treatment variables, and patient outcomes.

## 2 Patients and Methods

### 2.1 Patient Selection and Data Collection

All osteosarcoma subtypes in the COSS‐database had been classified according to the WHO‐classification [1]. The electronic COSS‐database was now searched for patients entered from 1980 to 2019 whose tumors represented the teleangiectatic variant.

Patients whose follow‐up ended within 100 days from diagnostic biopsy and before any attempt at local tumor control was made were excluded from this analysis unless they suffered events during this brief observation period.

COSS‐recruitment procedures and ‐treatment protocols have been reported in detail [3, 6, 7, 8]. Patient demographics, tumor characteristics, and therapy as well as follow‐up information were collected prospectively and coded as described [7]. Further information was derived from status report forms, radiology, pathology, and surgery reports, as well as progress letters, if available.

All COSS‐studies and ‐registry protocols were performed in accordance with the Code of Ethics of the World Medical Association (Declaration of Helsinki) and approved by the appropriate ethics committee (Ethik‐Kommission bei der Ärztekammer Hamburg nos. 500, 1147; Ethikkommission der Ärztekammer Westfalen‐Lippe und der Westfälischen‐Wilhelms Universität nos. 182/98 Biel2, 4IV Bie 2, 4 I Bielack, 5 V. Bielack; and Ethik‐Kommission an der Medizinischen Fakultät der Eberhard‐Karls‐Universität und am Universitätsklinikum Tübingen no. 5 V Bielack).

Pathologic diagnosis was made by histologic investigations and suitable immunohistochemistry techniques according to local practice. Reference pathology was recommended.

All COSS‐protocols called for conventional radiography of the tumor and the thorax and a whole‐body ^99^Tc‐methylene‐diphosphonate bone scan. Computed tomography, magnetic resonance tomography, and positron emission tomography were used according to era and availability.

Treatment was to follow the various guidelines and guidance detailed in the COSS‐protocols active at the time of patient enrolment. All registered patients were to receive pre‐ and post‐operative chemotherapy, the drug regimens varying over the years. They generally included high‐dose methotrexate, doxorubicin, and cisplatin. Many patients were also to receive ifosfamide, considerably fewer other agents.

Local treatment consisted of complete surgical resection, including removal of potential metastases when feasible. Surgical margins were recommended to be at least wide, according to the criteria established by Enneking et al. [9]. Tumor response to preoperative chemotherapy was graded according to Salzer‐Kuntschik et al. [10], with a 10% viable tumor cutoff between good and poor responders. A complete surgical remission was assumed if all known tumor foci were removed at least macroscopically.

Follow‐up guidelines included a search for potential local recurrences by conventional X‐ray (at q3 months for 4–5 years, only in case of clinical suspicion thereafter). Lung metastases were searched for by conventional chest X‐rays (q 4–8 weeks in years 1–2, q8‐12 weeks in 3–4, q6 months years 5–8 to 10). Later follow‐up was recommended, but performed as prescribed by the treating physician.

### 2.2 Statistical Analyses

All patients were evaluated on an intention‐to‐treat basis. Descriptive statistics were performed by the *χ* ^2^‐test. The date of osteosarcoma diagnosis was the starting date for all survival analyses. Overall survival was calculated until the date of death or last patient‐related information, whichever was appropriate. Event‐free survival was calculated until the date of first recurrence, the date last known to be alive, or the date of death without a recurrence, whichever was appropriate. Additional malignancies were noted but not counted as events.

Survival estimates were calculated using the Kaplan–Meier estimate with 95% confidence values [11]. Survival expectancies between unrelated cohorts were compared by the log‐rank test [12]. *p*‐values ≤ 0.01 were considered significant, and no correction for multiple testing was made. Statistical analyses were carried out using the SPSS statistical software (IBM Corp. Released 2022. IBM SPSS Statistics for Windows, SPSS version 29.0.0.0., Armonk, NY: IBM Corp.).

## 3 Results

### 3.1 Patients

Two‐hundred and twenty‐nine patients with a final diagnosis of teleangiectatic osteosarcoma were identified from the COSS‐database. Three of these were excluded from analysis because of being registered upon recurrence. Another three were excluded for loss of follow‐up within 100 days from biopsy and before any attempt at local control. This left 223 evaluable patients who form the basis of all further analyses (Germany [208], Austria [7], Switzerland [6], and Hungary [2]). These were registered by 103 different institutions (58 pediatric hospitals with 157, 45 non‐pediatric hospitals with 66 patients). A comparison of major findings made in this cohort with those from the literature is presented in Table 1.

**TABLE 1:** Teleangiectatic osteosarcoma in the literature.

	Huvos 1982 [2]^a^	Weiss 2007 [13]	Angelini 2016 [4]	Zhong 2023 [5]	Bielack 2025 (current series)
Recruitment	Single institution (MSKCC)	Single institution (St. Jude's)	Single institution (Rizzoli)	Registry (SEER)	Multiinstitutional (COSS)
	1921–1979	1978–2005	1985–2008	1989–2019	1980–2019
Number of patients	124	24	87	127	223
Age					
Median (range), years	16 (3–67)	15.7 (3.2–23)	16.6 (4.7–59.8)	ND	15.9 (3.7–69.7)
Mean	20 ± ND	20 ± ND	20 ± 15	18 ± 11	
Gender					
Male	74 (60%)	16 (67%)	47 (54%)	64 (50)	134 (60%)
Female	50 (40%)	8 (33%)	40 (46%)	63 (50%)	89 (40%)
Tumor site					
Extremity	109/124 (88%)	22 (92%)	84/87 (97%)	127/127 (100%)	208/223 (93%)
Metaphysis	100/109 (92%)	ND	74 (88%)	ND	200 (96%)
Diaphysis	8 (7%)	ND	8 (10%)	ND	7 (3%)
Small bones	1 (1%)	ND	2 (2%)	ND	1 (< 1%)
Trunk	10/124 (8%)	2/24 (8%)	3/87 (3%)	—	13 (6%)
Head & neck	3/124 (2%)	—	—	—	2 (1%)
Extraosseous	2/124 (2%)	—	—	—	—
Pathologic fracture					
None or none reported	86/122 (70%)	15/24 (63%)	60/87 (69%)	ND	157/223 (70%)
Present	36/122 (30%)	9/24 (38%)	27/87 (31%)	ND	66/223 (30%)
At diagnosis	ND	4/9 (44%)	ND	ND	45/66 (68%)
During treatment	ND	5/9 (56%)	ND	ND	21/66 (32%)
Extraosseous	2	—	—	—	—
Distant metastases at diagnosis					
Absent	ND	20 (83%)	77/87 (89%)	106/122 (87%)	197/223 (88%)
Present	ND	4 (17%)	10/87 (11%)	16/122 (13%)	26/223 (12%)
Unknown	ND	—	—	5	
Chemotherapy					
Pre‐ +/− postoperatively^b^	ND	14 (58%)	78/87 (90%)	66/73 (90%)	180/223 (81%)
Only after or without surgery	ND	10 (42%)	9/87 (10%)	7/73 (10%)	43/223 (19%)
None or sequence unknown	ND	—	—	54	
Number of chemotherapeutic agents administered					
< 3	ND	11/24 (46%)	14/78 (18%)	ND	2/221 (1%)
≥ 3	ND	13/24 (54%)	64/78 (82%)	ND	219/221 (99%)
No data	ND	—	9	ND	2
Type of surgery					
Amputation or RPL	ND	17 (74%)	15/86 (17%)	41/125 (33%)	57/215 (27%)
Resection	ND	5 (23%)	71/86 (83%)	84/125 (67%)	158/215 (73%)
No surgery	ND	2	1	—	8
Unknown	ND	—	–.	2	—
Response					
Good	9/11 (82%)	4 (33%)	62/78 (79%)	ND	117/165 (71%)
Poor	2/11 (18%)	8 (67%)	16/78 (21%)	ND	48/165 (29%)
No preop. chemotherapy, no surgery, or no data	113	12	9	127	58
Overall survival					
Follow‐up (range), years	ND	7.1 (1–23.7)	8 (5–22)^c^	ND	7.1 (0.2–32.1)
5 year overall‐survival	27%	67%	64%	ND	73%
10 year overall‐survival	ND	ND	61%	ND	66%

Abbreviations: COSS, Cooperative Osteosarcoma Study Group; MSKCC, Memorial Sloan Kettering Cancer Center; ND, no data; SEER, Surveillance, Epidemiology, and End Results Program.

^a^ Published prior to the WHO‐classification of bone tumors [1].

^b^ Includes patients who never proceeded to surgery.

^c^ Mean follow‐up with 25% confidence intervals.

Central pathology review was available for 164/223 (74%) cases. Patients not having been evaluated by reference pathology were older (*p* < 0.001) and more likely to have received surgery prior to starting chemotherapy (*p* = 0.009) but were otherwise comparable (Table 2).

**TABLE 2:** Patient and tumor characteristics.

	All patients	Reference pathology	Local pathology only	*p* (*χ* ^2^)
	223	164	59	
Age				
Median (min–max) years	15.9 (3.7–69.7)	15.3 (3.7–69.7)	17.1 (3.8–57.3)	
1st and 2nd decades of life	170 (76%)	134 (82%)	36 (61%)	**0.001**
3rd and later decades of life	53 (24%)	30 (18%)	23 (39%)	
Gender				
Male	134 (60%)	103 (63%)	31 (53%)	0.167
Female	89 (40%)	61 (37%)	28 (47%)	
Site of tumor origin				
Extremity	208 (93%)	153 (93%)	55 (93%)	0.985
Trunk or head & neck	15 (7%)	11 (7%)	4 (7%)	
Pathological fracture				
Absent	157 (70%)	115 (70%)	42 (71%)	0.878
Present	66 (30%)	49 (30%)	17 (29%)	
Before therapy	45	33	12	
During therapy	21	16	5	
Metastases at diagnosis				
Absent	197 (88%)	148 (90%)	49 (83%)	0.140
Present	26 (12%)	16 (10%)	10 (17%)	
Timing of surgery				
Surgery initially	34 (16%)	19 (12%)	15 (27%)	**0.009**
Preoperative chemotherapy	181 (84%)	140 (88%)	41 (73%)	
No definitive surgery	8	5	3	
Type of surgery				
Amputation or rotation‐plasty	57 (27%)	47 (30%)	10 (18%)	0.181
Limb‐saving resection	158 (73%)	112 (70%)	46 (82%)	
No definitive surgery	8	5	3	
Response to preoperative chemotherapy				
< 10% viable tumor cells	117 (71%)	89 (70%)	28 (74%)	0.668
≥ 10% viable tumor cells	48 (29%)	38 (30%)	10 (26%)	
Response unknown	15	12	3	
Primary surgery	35	20	15	
No definitive tumor‐surgery	8	5	3	
Macroscopic remission of all tumor‐sites				
Remission achieved	201 (90%)	150 (91%)	51 (86%)	0.267
Failure to achieve remission	22 (10%)	14 (9%)	8 (14%)	

*Note:* Comparison of teleangiectatic osteosarcomas assessed by reference pathology and those seen only locally. Bold value indicates *p* < 0.05.

At diagnosis, patients were a median of 15.9 (range: 3.7–69.7) and a mean of 18.1 ± 11.1 years old. Thirty‐three (15%) were in their first, 137 (61%) in their second, 27 (12%) in their third, and 13 (6%) in their fourth decade of life while 13 (6%) were even older. One hundred thirty‐four (60%) individuals were male, 89 (40%) female.

One patient was known to be affected by neurofibromatosis, its type not specified. Otherwise, there was no documented tumor predisposition syndrome.

Information on the duration of symptoms (pain and/or swelling) before diagnostic biopsy was available for 154/223 (69%) individuals. In these, it was a median of 56 (0–941) days. During this period, 10 patients were reported to have received surgery for a presumed benign bone tumor (three aneurysmal and three unspecified benign bone cysts, one chondroblastoma, one chondromyxoid fibroma, one osteochondroma, one hemangioma).

Teleangiectatic osteosarcomas were located in an extremity in 208/223 (93%; femur 115, tibia 41, humerus 37, fibula 9, radius 3, ulna 2, foot 1) cases, proximally (hip or shoulder) in 50/208 (24%) and further distally in 158/208 (76%). Only 7/208 (3%) of the extremity tumors involved a diaphysis. Thirteen/223 (6%; vertebrae 5, ilium 4, ribs 2, sternum 1, scapula 1) teleangiectatic osteosarcomas involved the axial skeleton and 2/223 (1%) the head and neck.

Pathologic fractures were recorded for 65/208 (31%) extremity tumors, 1/13 axial tumors (a rib‐lesion), and were absent in the head and neck, with 45 (68%) occurring before the start of chemotherapy and 21 (32%) thereafter.

Metastases at diagnosis were detected in 26/223 (12%) individuals. These involved the lungs in all 26 patients. Four/26 had additional osseous metastases, combined with metastases to distant muscles and distant lymph nodes in one of these, each.

All 223 (100%) patients received chemotherapy for a reported median period of 249 (1–461) days (1 duration unknown). It was administered without any definitive surgery in 8 (4%) individuals, exclusively pre‐operatively in 4/223 (2%), exclusively post‐operatively in 34/223 (15%), while 177/223 (79%) received both. Systemically active agents administered included doxorubicin in 219/221 (99%), high‐dose methotrexate in 210/221 (95%), cisplatin in 210/220 (95%), ifosfamide in 141/220 (64%), and other drugs in 41/220 (19%) (rest no data).

Local therapy included surgery of the primary tumor in 215/223 (96%) individuals. This was by amputation (38), rotation‐plasty (19), or limb‐salvaging resection (158). Among 200/208 (96%) operated extremity tumors, those 137/200 (69%) without a documented pathologic fracture proceeded to limb‐salvage in 104 (76%) cases. The 63/200 (32%) operated patients affected by pathologic fractures had limb‐salvage in 39/63 (62%) (34/44 (77%) of those with the fracture occurring before, 5/19 (26%) of those with it occurring during chemotherapy, *p* = 0.033, *χ* ^2^). Histologic response of the tumor to induction chemotherapy was reported for 165/215 (77%) while 35 had chemotherapy only post‐operatively and 15 had no response data. Response was good in 117/165 (71%) patients. Radiotherapy at a median dose of 51 (40–67) Gray (1 dose unknown) was administered to 10/223 (4%) primary tumors (7 in addition to, 3 instead of surgery). As a result of therapy, 201/223 (90%) achieved a macroscopically complete remission of all tumor sites, and 22/223 (10%) did not.

Follow‐up for events was a median of 5.8 (1 day—32.1) years from diagnosis. By then, 129/223 (58%) patients remained event‐free (median follow‐up 10.9 (0.3–32.1) years) and 94/223 (42%) developed events as defined. During follow‐up, a total of 5 further, unrelated malignancies (acute myeloid leukemia [AML], glioblastoma, melanoma, carcinoma of the kidney, unspecified mucinous neoplasm; not counted as events) arose. The events were: failure to achieve remission 22, osteosarcoma recurrences 68 (metastatic 57, local 7, combined 4), death of unrelated events 4 (1 each AML, melanoma, glioblastoma, cardiomyopathy). Among those 61 patients who suffered metastases, these affected the lungs in 54, distant bones in 9, lymph nodes in 2, and the pericardium in 1 (1 metastatic site(s) unknown, multiple mentions possible).

Median follow‐up for survival was 7.1 (0.2–32.1) years after diagnosis. It was 10.5 (0.2–32.1) years for 152/223 (68%) survivors (in osteosarcoma remission 137 [1st 129, 2nd 8]), alive with disease 15 (without achieving a remission 10, at recurrence 5 [1st 2, 2nd 1, 3rd 2]).

For the 71/223 (32%) deceased patients, median time to death was 2.5 (0.3–26.7) years from diagnosis. Causes of death were osteosarcoma 62 (without achieving a remission 11, at recurrence 51 [1st 24, 2nd 16, 3rd 6, 4th 5]), acute treatment complications (multi‐organ failure in 5th CR 1, secondary malignancy while in 1st osteosarcoma remission 3 (see above), and cardiomyopathy in 1st remission 1). The cause of death was not reported for four patients (1 each without having achieved CR, 2nd CR, 2nd and 6th recurrence).

**FIGURE 1:**
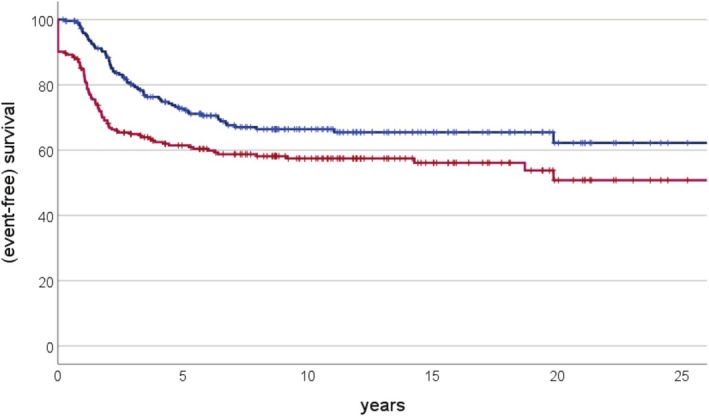
Event‐free (red) and overall (blue) survival estimates for all 223 patients diagnosed with teleangiectatic osteosarcoma.

Survival probabilities for all 223 patients at 1, 2, 5, and 10 years after diagnosis were 96% (1%), 88% (2%), 73% (3%), and 66% (3%) for overall and 85% (standard error 2%), 68% (3%), 61% (3%), and 57% (3%) for event‐free survival, respectively (Figure 1). Survival probabilities according to predictive and prognostic variables—again together with their distribution between those evaluated by reference pathology and those not—are presented in Table 3. For all patients, the presence of primary metastases (*p* < 0.001/*p* < 0.001, log‐rank test), occurrence of a pathologic fracture (*p* = 0.006/*p* = 0.002), and tumor response to neoadjuvant chemotherapy (*p* < 0.001/*p* < 0.001) were significant predictors of both event‐free and overall survival. The achievement of a complete surgical remission, only evaluable for overall survival, was also found to be significant (*p* < 0.001).

**TABLE 3:** Teleangiectatic osteosarcoma: overall and event‐free survival probabilities 5 years after histological diagnosis.

	All osteosarcomas					Reference pathology					Local pathology only				
	*n*	OS (%)	*p*	EFS (%)	*p*	*n*	OS (%)	*p*	EFS (%)	*p*	*n*	OS (%)	*p*	EFS (%)	*p*
All patients															
Total	223 (100%)	73% (3%)	—	61 (3)	—	164 (100%)	72 (4)	—	62 (4)	—	59 (100%)	74 (6)	—	61 (6)	—
Age at diagnosis															
≤ 15 years	112 (50%)	74 (4)	0.867	59 (5)	0.583	89 (54%)	72 (5)	0.776	58 (5)	0.651	23 (39%)	82 (8)	0.283	61 (10)	0.854
≥ 16 years	111 (50%).	72 (5)		64 (5)		0.75 (46%)	73 (5)		66 (6)		36 (61%)	69 (8)		60 (8)	
Gender															
Male	134 (60%)	74 (4)	0.731	63 (4)	0.705	103 (63%)	71 (5)	0.520	63 (5)	0.554	28 (47%)	62 (10)	0.043	65 (9)	0.758
Female	89 (40%)	70 (5)		58 (5)		61 (37%)	74 (6)		60 (6)		31 (53%)	86 (7)		55 (10)	
Tumor site															
Extremity	208 (93%)	73 (3)	0.337	63 (3)	0.055	153 (93%)	73 (4)	0.679	63 (4)	0.150	55 (93%)	75 (6)	*	63 (7)	*
Other	15 (7%)	64 (13)		37 (13)		11 (7%)	62 (15)		46 (15)		4 (7%)	*		*	
Metastases at diagnosis of osteosarcoma															
Present	26 (12%)	42 (10)	**< 0.001**	19 (8)	**< 0.001**	16 (10%)	47 (13)	**< 0.001**	25 (11)	**< 0.001**	10 (17%)	33 (16)	**< 0.001**	10 (10)	**< 0.001**
Absent	197 (88%)	77 (3)		67 (3)		148 (90%)	75 (4)		66 (4)		49 (83%)	82 (6)		71 (7)	
Pathological fracture															
None reported	157 (70%)	78 (3)	0.002	68 (4)	0.**006**	115 (70%)	79 (4)	0.002	71 (4)	**< 0.001**	42 (71%)	77 (7)	0.462	61 (8)	0.877
Documented	66 (30%)	60 (6)		46 (6)		49 (30%)	57 (7)		41 (7)		17 (29%)	69 (12)		59 (12)	
Start of therapy															
Surgery	34 (16%)	66 (8)	0.319	58 (9)	0.307	19 (12%)	66 (12)	0.621	56 (12)	0.442	15 (27%)	67 (12)	0.274	60 (13)	0.424
Chemotherapy	181 (84%)	75 (3)		65 (4)		140 (8%)	74 (4)		65 (4)		41 (73%)	79 (7)		65 (8)	
No surgery	8					5					3				
Tumor response															
Good	117 (71%)	86 (3)	**< 0.001**	78 (4)	**< 0.001**	89 (70%)	84 (4)	**< 0.001**	78 (5)	**< 0.001**	28 (74%)	89 (6)	**< 0.001**	78 (8)	**< 0.001**
Poor	48 (29%)	48 (8)		36 (7)		38 (30%)	51 (8)		39 (8)		10 (26%)	38 (17)		23 (14)	
No surgery or no response‐data	58					37					21				
Complete macroscopic surgical remission achieved															
Achieved	201 (90%)	76 (3)	**< 0.001**	68 (3)	—	150 (91)	75 (4)	**< 0.001**	68 (4)	—	51 (86%)	80 (6)	**< 0.001**	70 (7)	—
Not achieved	22 (10%)	30 (11)		NA		14 (9)	34 (14)				8 (14%)	20 (18)			

*Note:* Numbers in brackets represent 95%‐confidence intervals. *p*‐values below 0.01 are highlighted in bold.

Abbreviations: EFS, event‐free survival probabilities five years after diagnosis of osteosarcoma; OS, overall survival.

* indicates no calculation as *n* < 5.

## 4 Discussion

This large series of teleangiectatic osteosarcomas confirms many findings made by other, smaller analyses. These tumors affect sites outside of the extremities very rarely and seem to be exquisitely sensitive to chemotherapy. Prognostic factors resemble those of osteosarcoma in general. In line with findings from the EURAMOS trial [3], affected patients, particularly those with a good response to systemic treatment, may enjoy a better prognosis than other patients with osteosarcoma.

Our group primarily caters to the German‐speaking regions of Europe [6]. Based on population figures and overall osteosarcoma recruitment, we would have assumed that up to 10% of all cases originated in Austria. However, only 3% of teleangiectatic osteosarcomas in our registry were contributed from that country. There is no reason to believe this difference in frequencies to be real. Rather, the global gold standard for subtyping osteosarcomas, the WHO classification [1], seems to have left room for interpretation. It might benefit from further standardization.

It is undoubted that making a correct diagnosis of teleangiectatic osteosarcoma requires skill and expertise. Hence, we compared those almost three quarters of tumors for which centralized reference‐pathology was available with the rest. Apart from patients in the latter group being somewhat older and more prone to having received tumor surgery before starting systemic treatment, both cohorts seemed well comparable. The latter may be a result of the observed higher patient age: less experience with the disease among those caring for adults—where the disease is far less frequent—may have led to the use of treatments not current standard. Standard therapy may only have been administered when patients were in the hands of more experienced oncologists.

We cannot make any statements about the proportion of patients with teleangiectatic osteosarcomas who suffered previous malignancies. By definition, such tumors would be classified as secondary osteosarcomas [1]. Osteosarcomas in general seem to carry predisposing germline variants rather frequently [14]. It seems remarkable that we detected only a single case of known genetic tumor predisposition. This patient had neurofibromatosis, a syndrome not otherwise known to be associated with osteosarcomas [15]. While we can in no way be certain to have been informed of all cases of confirmed tumor predispositions, we still find the almost total lack of such reports quite intriguing.

The frequency of patients with secondary malignancies after teleangiectatic osteosarcomas was very similar to the one we recently reported for osteosarcoma in general [16]. Some of these secondary cancers may well have been treatment‐associated or due to coincidence rather than genetic predisposition. Other larger series remain largely silent regarding this topic [2, 4, 5, 13]. The same seems to hold true for the rest of the literature.

As in osteosarcoma in general [17] and in dedicated series of teleangiectatic tumors [2, 4, 5, 13], we observed most affected patients to be adolescents. However, as well as others [2, 4], we also observed teleangiectatic tumors arising in some older and even elderly patients. It therefore seems wise to be prepared for this rare diagnosis, whichever age the patient is.

As previously seen by others [2, 4, 13], we detected the slight male preponderance also typical for osteosarcoma in general. The interval between the first symptom and the diagnostic surgical procedure, approximately two months, again reflects the overall situation [18]. The same generally holds true for the distribution of teleangiectatic osteosarcomas in the skeleton [17]. Two peculiarities, however, seem remarkable: For one, craniofacial sites as well as those of axial bones, while present in our cohort, were detected infrequently. Also not assessed in other large published series, diaphyseal tumor locations seemed exceedingly rare. A tumor reported as teleangiectatic osteosarcoma which does not arise in the metaphysis of an extremity bone may therefore merit a closer look and second opinion.

It is most probably due to the highly osteolytic nature of this tumor that pathologic fractures were observed in almost one third of our patients. Similar observations have been made by others [2, 4, 13]. If a correct diagnosis is not made at the time of such a fracture, this may lead to erroneous surgical procedures which later force otherwise avoidable amputations. A considerable proportion of the pathologic fractures in our series and that from St. Jude's, the only other series reporting on the timing of this complication [13], occurred well after diagnosis when the patient was already receiving preoperative chemotherapy.

Osteosarcomas in general are associated with primary metastases in above 10%–20%. Neither our results nor those of others [4, 5, 13] point out that this is in any way different for the teleangiectatic variant. The same holds true for their predominantly pulmonary location.

All patients in our series received chemotherapy, the vast majority at least with three agents. This seems somewhat more intense than the average reported from other series [4, 13]. A relatively high proportion of therapies started with surgery rather than chemotherapy, often obviously necessitated by pathologic fractures. Similar observations have been published [4, 5, 13]. The duration over which systemic therapy was administered, a median of around eight months, is not different from other osteosarcomas [18]. This demonstrates that such treatment seems feasible.

Almost all our patients proceeded to surgery of the primary tumor. Like in other modern series on teleangiectatic osteosarcoma [4, 5], this was mainly by limb salvage. The occurrence of a pathologic fracture, however, was associated with a somewhat higher amputation rate, particularly if such only occurred once chemotherapy had been initiated.

The response‐rate to chemotherapy we observed, around 70%, was higher than that generally observed for osteosarcomas [17]. Similar findings of a high response‐rate were reported by the Rizzoli Institute [4]. It seems that teleangiectatic osteosarcoma may be a particularly chemosensitive disease variant. The pronounced flow of blood throughout the tumor and hence the accessibility of the malignant cells to the drugs employed may be one explanation. Too few patients in this series received radiotherapy to speculate about its efficacy.

This cohort is remarkable for its mature follow‐up, the median for survivors being just above 10 years. During this period, the type of tumor‐related events was not distinct from osteosarcoma in general. Failures were mainly pulmonary metastases and arose relatively early. Also, some local relapses and extrapulmonary metastases, particularly to distant bones, occurred. Primary metastases, pathologic fractures, and a poor tumor response to neoadjuvant chemotherapy were negative prognostic factors, as was failure to achieve a complete surgical remission. Remarkably, almost three‐quarters of our patients were still alive at five, and some two‐thirds even at ten years after diagnosis. In the large, intergroup EURAMOS study, teleangiectatic histology was found associated with the best prognosis [3]. Other series have also found survival rates of around two thirds [4, 13]. All these success rates seem somewhat higher than for those observed for high‐grade osteosarcoma in general [17].

In conclusion, teleangiectatic osteosarcoma is a variant of the disease that broadly resembles osteosarcoma in general. It does, however, come with its peculiarities. Familial predisposition may play a lesser role in the development than it does for other osteosarcoma variants. Tumors outside the extremities or those affecting their diaphyses seem very rare. Pathologic fractures, on the contrary, are quite frequent. Teleangiectatic osteosarcomas are often exquisitely chemosensitive. Current multimodal therapy may therefore cure more patients affected by this subtype than it does other osteosarcoma variants. Given that one third of patients still die within the first ten years, further attempts towards treatment optimization seem warranted.

## Author Contributions

**Stefan S. Bielack:** conceptualization (lead), data curation (equal), formal analysis (lead), investigation (equal), methodology (lead), project administration (equal), resources (supporting), validation (lead), visualization (equal), writing – original draft (lead), writing – review and editing (equal). **Vanessa Mettmann:** data curation (equal), validation (equal), writing – review and editing (equal). **Daniel Baumhoer:** data curation (equal), writing – review and editing (equal). **Andreas Beilken:** data curation (equal), writing – review and editing (equal). **Claudia Blattmann:** data curation (equal), funding acquisition (lead), resources (lead), software (equal), supervision (lead), writing – review and editing (equal). **Godehard Friedel:** data curation (equal), writing – review and editing (equal). **Jendrik Hardes:** data curation (equal), writing – review and editing (equal). **Wolf Hassenpflug:** data curation (equal), writing – review and editing (equal). **Leo Kager:** data curation (equal), writing – review and editing (equal). **Matthias Kevric:** data curation (equal), formal analysis (equal), writing – review and editing (equal). **Thekla von Kalle:** data curation (equal), writing – review and editing (equal). **Andreas Kulozik:** data curation (equal), writing – review and editing (equal). **Markus Metzler:** data curation (equal), writing – review and editing (equal). **Michaela Nathrath:** data curation (equal), writing – review and editing (equal). **Claudia Rossig:** data curation (equal), writing – review and editing (equal). **Benjamin Sorg:** data curation (equal), formal analysis (equal), writing – review and editing (equal). **Mathias Werner:** data curation (equal), writing – review and editing (equal). **Stefanie Hecker‐Nolting:** data curation (equal), investigation (equal), validation (equal), writing – review and editing (equal).

## Ethics Statement

All COSS‐study and ‐registry protocols were performed in accordance with the Code of Ethics of the World Medical Association (Declaration of Helsinki) and approved by the appropriate ethics committee (Ethik‐Kommission bei der Ärztekammer Hamburg nos. 500, 1147; Ethikkommission der Ärztekammer Westfalen‐Lippe und der Westfälischen‐Wilhelms Universität nos. 182/98 Biel2, 4IV Bie 2, 4 I Bielack, 5 V. Bielack; and Ethik‐Kommission an der Medizinischen Fakultät der Eberhard‐Karls‐Universität und am Universitätsklinikum Tübingen no. 5 V Bielack).

## Consent

Informed consent was required from all patients and/or legal guardians, whichever appropriate, before enrolment into the COSS‐database.

## Conflicts of Interest

Stefan S. Bielack reports consulting fees from MAP Biopharma, SERB SAS, Atheneum Partners GmbH, and Medicys Healthcare Fieldwork Experts, and payment for expert testimony by Zschimmer & Schwarz Mohsdorf GmbH & Co. KG. Daniel Baumhoer reports receiving support for the present manuscript from Basel Research Centre for Child Health. Jendrik Hardes reports grants from Implantcast Buxtehude, honoraria from Implantcast Buxtehude, support for attending meetings and/or travel from Implantcast Buxtehude, leadership or fiduciary role in other board, society, committee or advocacy group, in EMSOS and DGOOC. Michaela Nathrath reports leadership or fiduciary role in other board, society, committee or advocacy group, paid or unpaid, as lead of work package 1 of FOSTER (Fight Osteosarcoma Through European Reseach), co‐chair of COSS, and chair of the COSS biobank. Stefanie Hecker‐Nolting reports grants or contracts from DKKS, ERN PaedCan‐Y7‐Y10, and Förderverein krebskranke Kinder Stuttgart e.V., and leadership roles in FOSTER workpackage 3 (lead), HIBISCUS (co‐chair), and the Cooperative Osteosarcoma study group COSS of GPOH (chair). Claudia Rossig reports honoraria from Amgen, honoraria from Novartis, and attending an Advisory Board hosted by Vertex Pharmaceuticals.

Andreas Beilken, Claudia Blattmann, Godehard Friedel, Wolf Hassenpflug, Leo Kager, Matthias Kevric, Thekla von Kalle, Andreas Kulozik, Markus Metzler, Vanessa Mettmann, Benjamin Sorg, and Mathias Werner report that they have no conflicts of interest.

## Data Availability

The data that support the findings of this study are available from the corresponding author upon reasonable request.
